# A comparison between survival from cancer before and after a physical traumatic injury: physical trauma before cancer is associated with decreased survival

**DOI:** 10.1186/s13032-015-0029-y

**Published:** 2015-11-04

**Authors:** Douglas L. Delahanty, Robert Marley, Andrew Fenton, Ann Salvator, Christina Woofter, Daniel Erck, Jennifer Coleman, Farid Muakkassa

**Affiliations:** Kent State University, Kent, Ohio USA; Northeast Ohio Medical University, Rootstown, Ohio USA; Akron General Medical Center, Akron, Ohio USA; West Virginia School of Osteopathic Medicine, Lewisburg, West Virginia USA; Trauma and Surgical Intensive Care Unit, Akron General Medical Center, 1 Akron General Avenue, Akron, OH 44307 USA

**Keywords:** Physical trauma, Injury, Cancer, Prognosis, Survival

## Abstract

**Background:**

Prior traumatic experiences have been associated with poorer coping strategies, greater distress, and more posttraumatic stress disorder (PTSD) symptoms following a subsequent cancer diagnosis affecting their survival. However, the impact of prior physical traumatic injury on cancer survival has not been examined.

**Methods:**

The present study matched patients from the same Level 1 Trauma center who appeared in both the trauma and cancer registries. A total of 498 patients met the criteria between 1998 and 2014 who have experienced both a diagnosis of cancer and a physical traumatic injury. The survival between the patients who had physical trauma before cancer (TBC) versus those that had physical trauma after the cancer diagnosis (TAC) were compared.

**Results:**

The TBC group had a higher percentage of males (48 % vs 33 % *p* = 0.001) and motor vehicle collisions (18 % vs 7 %, *p* < 0.001), than the TAC group. TBC patients were also significantly younger than TAC patients at the time of the physical traumatic event (68.7 ± 14.6 vs 76.2 ± 12.0 years, *p* < 0.001), and longer length of time between the cancer diagnosis and physical traumatic injury (2.9 ± 2.9 vs 1.7 ± 2.6 years, *p* < 0.001). The overall probability of survival for the entire sample was 68 %. Percent survival for the TBC (*n* = 251) and TAC (*n* = 247) groups was 56 and 80 % respectively (*p* < 0.001).

Results were consistent regardless of stage of cancer at diagnosis (hazard ratio (HR (Standard Error)). After adjusting for comorbidities Charlson comorbidity index (CCI) (HR = 1.2 (0.06), *p* = 0.009)), cancer stage (HR = 2.8 (0.12), *p* < 0.001)), lung cancer (HR = 1.7 (0.25), *p* < 0.001) and bladder cancer (HR = 3.5 (0.55), *p* = 0.02), experiencing a prior physical traumatic injury was associated with an increased HR for mortality of 4.6 (0.93), *p* < 0.001).

**Conclusions:**

A physical traumatic episode before cancer diagnosis (TBC) increased the risk of death 4.6 fold compared to the TAC group even after adjusting for CCI, stage of cancer at diagnosis, lung cancer, and bladder cancer. These findings suggest considering a history of physical traumatic injury in cancer patients as a possible risk factor for faster cancer progression and mortality.

## Background

The term “cancer” refers to a heterogeneous group of malignant neoplasms characterized by uncontrolled and rapid cell growth and the potential to invade surrounding tissue and metastasize to new sites. Different types of cancer vary dramatically with regards to etiology, disease course, and treatment [1]. Therefore, it has been difficult to elucidate specific factors that increase risk for mortality across different types of cancer. However, a number of factors have been found to impact patient outcome in terms of mortality and disease-free intervals across most cancers including: (1) age, (2) genetics/heredity, (3) initial stage of cancer at detection, and (4) host/behavioral factors [2–4].

One potential risk factor that has not been examined is a history of physical traumatic injury. The fifth edition of the Diagnostic and Statistical Manual of Mental Disorders (DSM-5: American Psychiatric Association, 2013) [5] defines a potentially traumatic stressful event as one that directly or indirectly exposes a person to “death, threatened death, actual or threatened serious injury, or actual or threatened sexual violence”. Physical traumatic injury is a common occurrence in America, resulting in over 41 million emergency department visits and 2.3 million hospital admissions yearly. Further, injuries stemming from road traffic crashes alone have been projected to be the third largest global burden of disease by 2020 [6]. Mental health outcomes following physical traumatic injury range from transient distress to a diagnosis of posttraumatic stress disorder (PTSD) and other comorbid disorders. A nationwide study found that 23 % of injury patients met PTSD diagnostic criteria 12 months post-injury [7], and 18 % of traumatic injury patients continued to have poor recovery trajectories 6-years post-injury [8]. However, the impact of physical trauma history on mortality from cancer has not been examined. The present study represents an initial examination of the relationship between history of physical traumatic injury and cancer mortality. It focuses on history of physical traumatic injury as traumatic events involving physical injury consistently result in higher rates of PTSD symptoms than those that do not involve injury [9], and because history of physical traumatic injury was objectively verifiable (compared to other traumas that typically rely on self-report). This study hypothesized that cancer patients with a documented prior physical traumatic injury would have lower survival rates than cancer patients without such history.

## Methods

Patients who could objectively be identified as having experienced both a cancer diagnosis and an injury requiring treatment at an American College of Surgeons verified Level 1 Trauma Center were identified using both the Level I Trauma registry from 1998 to 2014 and the Cancer registry from 1981 to 2013. The range of dates in the trauma registry was chosen to correlate with the date of verification of the Level 1 Trauma Center till when this study was ended in 2014. The cancer registry data range was chosen from the inception of the cancer registry at the trauma center up to the year 2013 when complete data was available for the current study. Therefore, any patients in the trauma registry in the year 2014, would have only been included in the trauma after cancer group. The trauma registry only includes patients admitted to the hospital due to the severity of their injuries and not patients treated and released from the Emergency Department. The patients were matched by medical record numbers from both registries. To avoid duplication of data when patients were admitted multiple times, the trauma admission closest to the cancer diagnosis was utilized. Both the trauma and cancer registries collect data prospectively in a timely fashion on all cancer and trauma patients admitted to the Level 1 trauma center by trained registrars and adhere to quality standards as set by states and national organizations. The trauma registry collects data describing etiologic factors, demographic characteristics, diagnoses, treatments and clinical outcomes of the trauma patients. The cancer registry captures a complete summary of the patient’s history, diagnosis, treatment, and status for every cancer patient with in situ or invasive cancers with histologically proven tumors (no benign tumors). Mortality from the cancer registry is determined by several sources including regular monitoring of the patient’s chart, monthly death match from the Social Security Death Index and a monthly death list from the State records. Only the cancer deaths verified from all these sources were defined as deaths in the survival analysis. Those patients in the cancer registry whose death could not be attributed to their cancer or unknown were censored (*n* = 67). Patients who died from the traumatic injury during the index hospitalization were also censored (*n* = 6).

As pre-existing medical conditions have been associated with elevated mortality rates of 20 % in cancer patients [10] and increased relative odds ratios for mortality of 1.8 [11] in physical trauma patients, these conditions are routinely assessed as part of a trauma admission. However, cancer registries do not routinely record prior physical traumatic injury as part of their data set. Thus, it was impossible to determine whether patients appearing only in the cancer registry had or had not experienced a prior injury necessitating treatment at a trauma center. This limited our ability to match cancer patients with a physical traumatic injury history to those without a physical traumatic injury history. Therefore, the study used as controls cancer patients who experienced a physical traumatic injury *after* their cancer diagnosis. This further allowed the examination of whether survival differences were simply due to the presence of a physical trauma, or whether the temporal ordering of physical trauma and cancer was differentially related to survival (i.e., whether survival differences existed between cancer patients who experienced a physical traumatic injury prior to diagnosis and cancer patients who experienced a physical traumatic injury after the diagnosis).

A total of 498 patients appeared in both registries (203 men and 295 women; mean age = 72 years; age range = 20–101 years). Two hundred fifty-one patients were identified as having experienced a physical traumatic injury requiring hospitalization before they were diagnosed with cancer (TBC), and 247 patients were identified as having experienced a physical traumatic injury requiring hospitalization after being diagnosed with cancer (TAC). In order to examine whether physical traumatic injury impacted survival relative to cancer patients without documented physical traumatic injury, a control group comprised of cancer patients who appeared in the cancer registry but not in the trauma registry during the study period (1981–2013) were analyzed as well (CAreg group, *n* = 7470).

A number of factors previously shown to predict survival were obtained from the registry databases. These included demographic data (age and gender), cancer-related data (type and stage of cancer at diagnosis, age at cancer diagnosis, length of survival and time between cancer diagnosis and trauma), and trauma-related data (age at trauma, type of trauma, whether the patient received a transfusion of packed red blood cells (PRBC) during the physical trauma hospitalization, history of surgery, and Injury Severity Score (ISS)) [12]. Further, to determine whether presence of disease comorbidities other than cancer impacted survival, the Charlson Comorbidity Index (CCI) was calculated according to published guidelines [13] and included in the analyses to control for the effect of comorbidities on the mortality of the TBC and TAC groups.

Packed red blood cell transfusions (PRBC) were reviewed as a control variable as such transfusions have been associated with decreased survival in some studies of patients with colorectal cancer [14], and have a dose-dependent effect on enhancing growth of MC7 sarcomas in rats [15]. A study by Kamper-Jørgensen showed that 3 months after the first transfusion, 84.3 % of recipients were alive [16]. In addition the study showed that after 1-, 5- and 20-years, post-transfusion survival was 73.7, 53.4 and 27.0 %, respectively, thus emphasizing the long term effects of blood transfusion on survival.

A history of surgical procedures was also examined as a possible control variable as undergoing surgery and receiving anesthesia has been found to be immunosuppressive and to contribute to tumor growth and metastasis [17, 18]. Although the exact mechanism is not known, some researchers have suggested that it could be due to surgically mediated decrease in NK cell activity) [19].

Lastly we examined whether the severity of the physical traumatic injury may have affected mortality. Injury Severity Score is an accepted standard in the USA to measure severity of injury and have been found to correlate linearly with mortality, morbidity, hospital stay and other measures of severity. The ISS is an anatomical scoring system calculated from the Abbreviated Injury Scale (AIS [20]) score that looks at six body regions (head, face, chest, abdomen, extremities (including pelvis), and external). The extent of injury to each region is ranked on a scale from 0 to 6, with 0 = none, 1 = minor, 2 = moderate, 3 = serious, 4 = severe, 5 = critical and 6 = unsurvivable injury. The three most severely injured body regions have their scores squared and summed to produce the ISS. The ISS ranges from 1 to 75 with a score more than 15 indicating a major trauma. Therefore an ISS of 15 was utilized in this study as a cut-off point to compare severity of trauma between the TBC and TAC groups.

Twenty-eight patients were diagnosed with cancer as an incidental finding during their workup at their index hospitalization for their physical traumatic injuries. These patients were placed in the TAC group because their undiagnosed cancer existed before the physical trauma.

### Statistical analyses

The probability of cancer survival was estimated using the Kaplan-Meier method with the log rank test to estimate differences among levels of the analyzed variables. Length of cancer survival was defined as the time interval from diagnosis of a cancer to death from cancer. Patients who died of causes other than cancer were censored in the analyses. However, inclusion or exclusion of these censored subjects did not change the findings.

A Cox proportional hazard model was examined by assessing all factors that significantly predicted survival in univariate models (*p* < 0.05) for joint prognostic value in a multivariate model. The reduced model was developed using a backward selection strategy. All model assumptions were met for the analysis. Patients were next classified into groups depending on their stage of cancer at diagnosis per the American Cancer Society criteria, and analyses were repeated separately for each stage. Statistical analyses were performed using SPSS software version 22.0 (SPSS Inc., Chicago, IL, USA). The Institutional Review Board and Human Subjects Review Committee at the hospital approved the study as an exempt study.

## Results

The TBC group had a higher percentage of males (48 % vs 33 % *p* = 0.001) and motor vehicle collisions (MVC) (18 % vs 7 %, *p* < 0.001), than the TAC group. TBC patients were also significantly younger than TAC patients at the time of the physical traumatic event (68.7 ± 14.6 vs 76.2 ± 12.0 years, *p* < 0.001), had lower ISS scores (7.2 ± 4.6 vs 8.3 ± 5.0 years, *p* = 0.01), and longer length of time between the cancer diagnosis and physical traumatic injury (2.9 ± 2.9 vs 1.7 ± 2.6 years, *p* < 0.001). The TBC group also had a significantly lower percentage of physical trauma-related PRBC transfusions (14 % vs 23 %, *p* = 0.03) and medical anemia-related PRBC transfusions (3 % vs 8 %, *p* = 0.02). There were no differences between the groups with respect to the number of physical trauma-related surgeries during the hospital admission (40 % vs 44 %, *p* = 0.28). The TBC group had significantly fewer comorbidities (according to the CCI) than the TAC group (CCI = 2.98 ± 1.8 vs 3.91 ± 1.7, *p* < 0.001) (Table 1).

**Table 1:** Differences between trauma before cancer (TBC) and trauma after cancer (TAC) groups

	TBC (*n* = 251)	TAC (*n* = 247)	*p*-value
Age (years) at time of Traumatic injury	68.7 ± 14.6	76.2 ± 12.0	<0.001
Age (years) at time of cancer diagnosis	71.6 ± 14.4	73.22 ± 12.1	0.18
% Male	121 (48 %)	82 (33 %)	0.001
Injury Severity Score (ISS)	7.2 ± 4.6	8.3 ± 5.0	0.01
% Fall	162 (67 %)	207 (87 %)	<0.001
% Motor Vehicle Crash (MVC)	43 (18 %)	17 (7 %)	<0.001
Charlson Comorbidity Index (CCI)	2.98 ± 1.8	3.91 ± 1.7	<0.001
% Trauma Related Surgery	96 (40 %)	106 (44 %)	0.28
% Transfused PRBC due to trauma	36 (15 %)	54 (23 %)	0.03
% Transfused PRBC due to anemia	7 (3 %)	18 (8 %)	0.02
% Died from physical trauma	2 (1 %)	4 (2 %)	<0.001
% Died from Cancer	126 (50 %)	57 (23 %)	<0.001
Survival time from cancer diagnosis (years)	2.0 ± 2.0	4.5 ± 2.5	<0.001
Time between trauma and cancer diagnosis (years)	2.9 ± 2.9	1.7 ± 2.6	<0.001

**Fig. 1:**
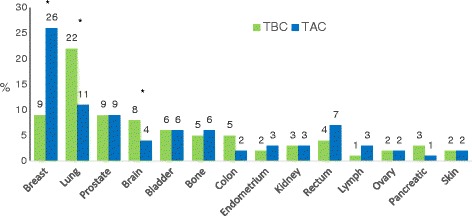
Difference in Cancer Types (%) Between TBC and TAC. Legend: *TBC* Trauma Before Cancer, *TAC* Trauma After Cancer. *Indicates a statistically significant difference between TBC and TAC

Differences between TBC and TAC regarding type of cancers are illustrated in Fig. 1 in descending order of frequency. The TBC group had a statistically higher incidence of lung (22 % vs 11 %, *p* = 0.001) and brain cancer (8 % vs 4 %, *p* = 0.04) compared to the TAC group and a statistically lower incidence of breast cancer (9 % vs 27 %, *p* < 0.001).

The overall probability of survival for the entire sample was 68 %. Percent survival for the TBC and TAC groups was 56 and 80 % respectively, with the difference between survival curves being statistically significant (*p* < 0.001).

**Fig. 2:**
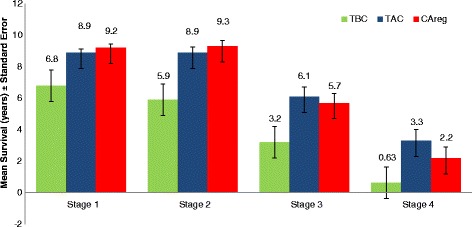
Difference in Mean Survival (years) Between TBC, TAC and CAreg by Cancer Stage. Legend: *TBC* Trauma Before Cancer, *TAC* Trauma After Cancer, *CAreg* Cancer Registry

**Fig. 3:**
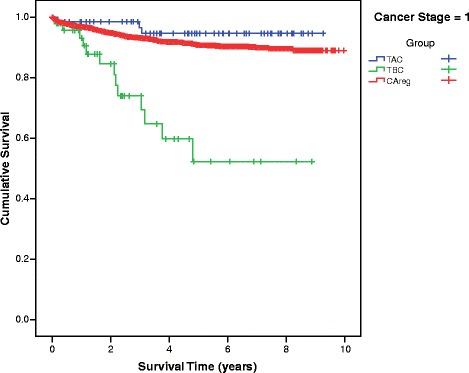
Survival Curves by TAC, TBC and CAreg in Stage 1 Cancer. Legend: *TBC* Trauma Before Cancer, *TAC* Trauma After Cancer, *CAreg* Cancer Registry

**Fig. 4:**
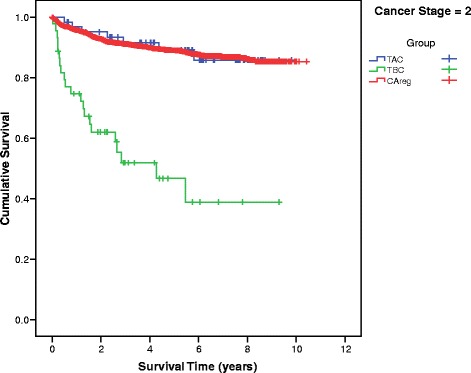
Survival Curves by TAC, TBC and CAreg in Stage 2 Cancer. Legend: *TBC* Trauma Before Cancer, *TAC* Trauma After Cancer, *CAreg* Cancer Registry

**Fig. 5:**
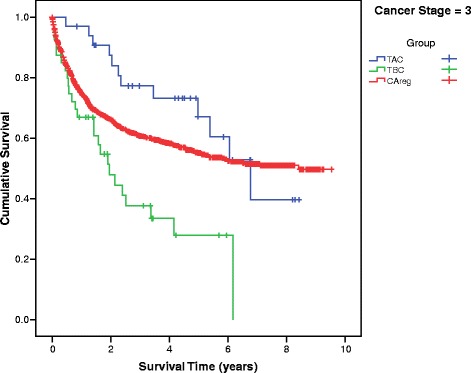
Survival Curves by TAC, TBC and CAreg in Stage 3 Cancer. Legend: *TBC* Trauma Before Cancer, *TAC* Trauma After Cancer, *CAreg* Cancer Registry

**Fig. 6:**
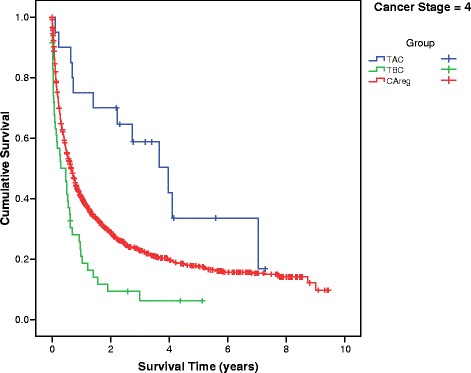
Survival Curves by TAC, TBC and CAreg in Stage 4 Cancer. Legend: *TBC* Trauma Before Cancer, *TAC* Trauma After Cancer, *CAreg* Cancer Registry

Differences in mean survival time between the TBC, TAC and CAreg groups were significant across patients at every stage of cancer at diagnosis (Fig. 2). The survival curves of the TBC, TAC and CAreg groups for each cancer stage (1–4) are illustrated in Figs. 3, 4, 5 and 6.

We next stratified the data by ISS (ISS < 15 = mild/moderate injury and ISS ≥15 = severe injury) to see if the severity of injury impacted mortality from the cancer between the TBC and TAC groups (Table 2). The mean survival (in years) did not differ between the TBC and TAC groups regardless of whether the physical traumatic injury was mild/moderate or severe.

**Table 2:** Survival analysis stratified by Severity of Physical trauma

	TBC	TAC	*p*-value
Survival time from cancer diagnosis (years)	2.0 ± 2.0 (*n* = 241)	4.5 ± 2.5 (*n* = 238)	<0.0005
Survival time (ISS < 15)	2.0 ± 1.9 (*n* = 187)	4.4 ± 2.6 (*n* = 177)	<0.0005
Survival time (ISS > = 15)	2.3 ± 2.4 (*n* = 41)	4.8 ± 2.5 (*n* = 53)	<0.0005

A multivariate Cox Proportional Hazard Model was next performed to determine the extent to which identified prognostic factors affected survival in the entire sample. Although they had been associated with survival in univariate analyses, gender (female = 1, male = 0), ISS, prostate cancer (1 = yes, 0 = no), brain cancer (1 = yes, 0 = no), breast cancer (1 = yes, 0 = no), age of cancer diagnosis, age of physical trauma and cancer grade were not associated with increased risk of mortality in the multivariate model (*p* < 0.10) and were eliminated. According to the final reduced model, five variables (CCI, cancer stage, lung cancer, bladder cancer and TBC) were statistically significant predictors of mortality. Experiencing a physical traumatic event prior to the cancer diagnosis (TBC) increased the risk of death more than 4 fold (HR = 4.6 (0.93), *p* < 0.001) even after adjusting for CCI (HR = 1.2 (0.06), *p* = 0.007), stage of cancer at diagnosis (HR = 2.0 (0.17), *p* < 0.001), lung cancer (HR = 2.1 (0.41), *p* < 0.001), and bladder cancer (HR = 3.2 (1.1), *p* = 0.001).

The 28 patients who had their cancer diagnosed during their index hospitalization and patients who had their cancer diagnosed within 12 months of their index hospitalization (*n* = 63), present a special group of cancer patients where the duration of their cancer is unknown. This may have introduced a bias in their classification in the TAC group. To address and test this potential bias, these 91 cases were placed in the TBC group and a sensitivity model was run. The results of experiencing a physical traumatic event prior to the cancer diagnosis was then increased 5.4 fold (HR = 5.4 (0.30), *p* < 0.001) after adjusting for CCI, stage of cancer, lung cancer and bladder cancer. This validated our initial classification of the TAC group as it made the results even more significant (5.4 vs 4.6 fold). At the end, the classification of cases where patients had cancer as an incidental finding or within 12 months of their physical trauma were kept in the TAC group in the final analysis.

## Discussion

The present study was designed to examine the relationship between physical traumatic injury and mortality from cancer in cancer patients. The results supported this study hypotheses in that patients with a prior history of physical traumatic injury had lower survival rates relative to the cancer patients who experienced a physical traumatic injury after their cancer diagnosis. Differences in survival related to temporal ordering of physical trauma and cancer diagnosis were consistent regardless of the stage of cancer at diagnosis. In the present study, patients who experienced a physical traumatic injury after their cancer diagnosis had a significantly higher survival rate than patients who experienced a similar injury before diagnosis. This was true even though TAC patients were significantly older at the time of the physical trauma and had significantly more PRBC transfusions, underscoring the importance of considering physical trauma history as a risk factor for faster cancer progression and mortality. Further, the observed survival differences were not due to other risk factors such as whether or not the trauma involved an operation, severity of physical injury, or presence of disease comorbidities.

Blood transfusions have been associated with immune suppression in some studies and increase death in others even after years of the initial transfusion [15, 16]. This study examined whether there were any statistical differences in the transfusion of PRBC between the TBC and TAC groups during their index hospitalization that could have accounted for or affected the mortality between the groups but none was found. This was true if the PRBC were transfused secondary to acute blood loss from the traumatic injuries or due to medical causes of anemia for which blood was transfused.

Another factor studied to assess the impact on survival was operative interventions and the stress they induce on the patients, being physical, chemical or emotional [21]. In this study there was no difference in the number of operations performed due to the physical traumatic injuries between the TBC and TAC groups. In this regards, this retrospective study could not discern whether surgical stress could have contributed to the difference in survival between the TBC and TAC groups.

It is also reasonable to hypothesize that the more severe the physical injury, as measured by the ISS score, the more stress (physical or otherwise) the body will suffer and hence the higher the mortality from cancer. Such association was not found in this study when the patients were stratified to mild/moderate injury (ISS < 15) or severe injury (ISS ≥ 15). There was no statistical difference in survival time from cancer diagnosis between the TBC and TAC groups regardless of severity of injury.

Although there was no statistical difference in age at time of cancer diagnosis between the TBC and TAC groups, it was surprising to note that patients with TBC, with shorter survival than those with TAC, tended to be younger at the time of their physical trauma. One would expect to find a higher mortality from cancer in elderly patients due to their age and comorbidities. Contrary to our expectations, the TBC group had a lower CCI yet had decreased survival compared to the TAC group who had a higher CCI. Comorbidities did not explain the higher mortality rate in the TBC group in this study.

There was a difference in the mechanism of injuries between the TBC and TAC groups. This can be explained by the fact that, in general, younger, male patients are involved more in MVC compared to the older female population who are more prone to falls. Despite the older population, female gender and falls as a mechanism of injury in the TAC group, their survival was better than the younger, MVC and male cancer patients in the TBC group.

When looking at the major cancer types between the TBC and TAC groups, there were a statistically significant increase number of lung and brain cancers in the TBC group and a statistically significant increase number of breast cancers in the TAC group. One explanation of the TBC group shorter survival may be explained by the increased number of the more aggressive lung and brain cancers. However when adjusting for both cancers and stage in the multivariate model, TBC still remained a significant predictor of mortality. The TAC group had more elderly female patients which explains the increased incidence of breast cancer in this group. One could hypothesize that the slower growing breast cancers in the TAC group could have accounted for the longer survival rate compared to the TBC group. However breast cancer was not a significant predictor in the multivariate model when adjusting for breast cancer stage.

As the present methodology relied on retrospective analysis of registry records, it was impossible to examine potential mechanisms (other than those included in the respective registries) for the observed differences in survival rates between TBC and TAC patients. Numerous psychosocial variables may account for the observed differences in survival. For instance, prior research has found that gastric cancer patients who reported a previous physical trauma also experienced greater depressive symptoms, less social support, and lower future life satisfaction [22]. Depression, in particular, has been associated with increased risk for developing and dying from cancer. In addition, PTSD symptoms are relatively common following a serious traumatic injury and are often comorbid with other mental health symptoms [7, 23]. PTSD is also associated with a number of negative effects including poor quality of life [24], and increased suicidality [25]. Numerous studies have suggested that increased stress and depression are associated with decreased quality of life, decreased length of survival, and shorter disease-free intervals in cancer patients [26]. Future, prospective studies should examine psychosocial (e.g., stress and depression) symptoms as possible mediators of the relationship between physical traumatic injury history and cancer survival.

A number of biological variables may also be associated with faster disease progression in cancer patients with a history of physical trauma. Chronic stress has been reliably associated with suppression of natural killer (NK) cell activity, and lower NK activity has been associated with shorter disease-free intervals and shorter length of survival in breast cancer patients [19]. Similarly, recent research has suggested that pro-angiogenic cytokines (e.g., vascular endothelial growth factor (VEGF)) may serve a key role in the pathogenesis of ovarian cancer [27]. Psychosocial stress and stress hormones stimulate production of pro-angiogenic cytokines [28], enabling formation of new blood vessels and enhancing tumor growth. Although it is unknown why these biological variables may be differentially impacted in cancer patients who differ in the temporal ordering of cancer diagnosis and trauma, future research should consider the inclusion of these stress- and disease-related biological measures.

Limitations associated with retrospective review of registry data include the inability to control for type and duration of cancer, physical trauma treatment or whether the TBC or TAC patients may have suffered an additional injury that was treated in a different facility. Other limitations may include the socioeconomic status (SES) of the patients which the trauma and cancer registries do not collect. As the study hospital is a Level 1 Trauma Center and a not-for-profit hospital, all patients admitted are treated equally regardless of their SES status and as such, there is no reason to believe that TBC and TAC groups received different treatments. However, all cancer and physical trauma treatments were conducted in the same medical facility, and there is no reason to believe that TBC and TAC patients differed in treatment received. Despite these limitations, the present study suggests that temporal ordering of physical traumatic injury and cancer diagnosis may be associated with differential survival rates in patients who experience both a cancer diagnosis and physical traumatic injury Survival differences were consistent regardless of stage of cancer at diagnosis Although future prospective studies are necessary to elucidate possible mechanisms for these relationships, the present findings suggest considering physical trauma history in cancer patients as a possible risk factor for faster cancer progression and mortality.

## Conclusion

A physical traumatic episode before cancer diagnosis (TBC) increased the risk of death 4.6 fold compared to the TAC group even after adjusting for CCI, stage of cancer at diagnosis, lung cancer, and bladder cancer. It is recommended that the inclusion of history of physical trauma be captured by cancer registries to further shed light on this association between TBC and decreased survival time in cancer patients.
